# Ferroquine, an Ingenious Antimalarial Drug –Thoughts on the Mechanism of Action

**DOI:** 10.3390/molecules13112900

**Published:** 2008-11-20

**Authors:** Faustine Dubar, Jamal Khalife, Jacques Brocard, Daniel Dive, Christophe Biot

**Affiliations:** 1 Université des Sciences et Technologies de Lille, Unité de Catalyse et Chimie du Solide – UMR CNRS 8181, Ecole Nationale Supérieure de Chimie de Lille, Bâtiment C7, B.P. 90108, 59652 Villeneuve d' Ascq cedex, France; E-mails: faustine.dubar@free.fr (F. D.), jacques.brocard@univ-lille1.fr (J. B.); 2 Inserm U547, Institut Pasteur, 1 rue du Pr Calmette, B.P. 245, 59019 Lille Cedex, France; E-mails: jamal.khalife@pasteur-lille.fr (J. F.), daniel.dive@pasteur-lille.fr (D. D.)

**Keywords:** Malaria, Bioorganometallics, Ferroquine, Mechanism of action, Resistance

## Abstract

Ferroquine (FQ or SR97193) is a novel antimalarial drug candidate, currently in development at Sanofi-Aventis. In contrast to conventional drugs, FQ is the first organometallic drug: a ferrocenyl group covalently flanked by a 4-aminoquinoline and a basic alkylamine. FQ is able to overcome the CQ resistance problem, an important limit to the control of *Plasmodium falciparum*, the principal causative agent of malaria. After fifteen years of effort, it is now possible to propose a multifactorial mechanism of action of FQ by its capacity to target lipids, to inhibit the formation of hemozoin and to generate reactive oxygen species.

## Introduction

**Figure 1:**
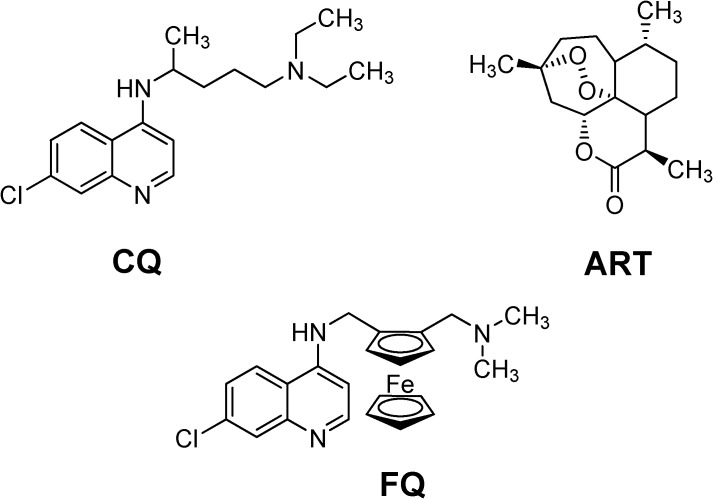
Chemical structures of chloroquine (CQ), artemisinin (ART) and ferroquine (FQ).

Malaria is a tropical disease causing almost three million deaths every year, mainly among children and pregnant women in Africa and South East Asia [1]. It is caused by single cell protozoon parasites of the *Plasmodium* species, of which *P. falciparum* is the most dangerous and accounts for 90% of all deaths from malaria. All of the quinoline-based compounds currently marketed (such as chloroquine, CQ, Figure 1) encounter chemoresistance problems [2]. Artemisinin (ART) derivatives represent a promising curative alternative for the future but their thermal instability [3,4] and the high cost of therapy [5] will limit their use in countries where access to the treatment is difficult. Combined therapies comprising of long acting (quinoline) and short acting ART-derived drugs offer efficient but expensive treatments. Furthermore, efficient vaccines will not be available in the near future [6].

This article focuses mainly on the pharmacology and the mechanism(s) of action of ferroquine (FQ, SAR97193), a new antimalarial, including its prospects in antimalarial therapy. In the mid 1990s, inspired by the impressive works of Pr. Gérard Jaouen in cancer chemotherapy [7,8], we applied the bioorganometallic strategy of analogue generation to the antimalarial class. As of today, more than 100 ferrocene analogues have been synthesized and screened [9,10,11], FQ proved to be the best antimalarial candidate. FQ is being developed by Sanofi-aventis and entered phase II clinical trials in September 2007 [12]. This is more amazing when we consider that FQ was the first analogue synthesized during the project. FQ meets with the Lipinski's ‘Rule of 5’ which orally active drugs have to follow to be efficient. Recently, relation-structure activities have revealed that, in addition to the known pharmacophores, the ferrocenyl moiety has to be as an integral part of the side chain of CQ [13].

## Specific pharmacology

**Figure 2:**
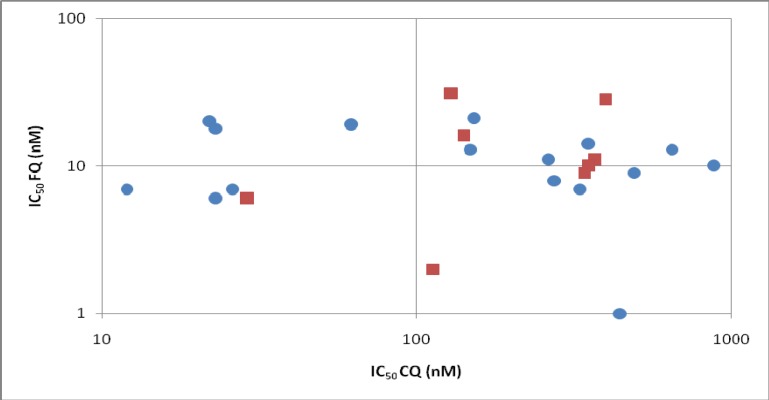
Comparison of *in vitro* sensitivity to CQ and FQ on 16 different laboratory *P. falciparum* clones (blue circles) and on eight sets of field isolates from Gabon, Senegal, and Cambodia (red squares).

*In vitro*, FQ has been tested on 16 different laboratory *P. falciparum* strains [14,15,16,17] and on eight sets of field isolates (total 441) from Gabon, Senegal, Cambodia and Thailand [18,19,20,21,22,23]. Figure 2 shows the mean IC_50_ observed from these different studies. In some cases, when tested on field isolates from different geographic origins, FQ showed a slight cross response with CQ [18,19,20,21,23], but it was weak and, in mind of authors, did not have a clinical significance for a cross-resistance between the drugs. Moreover, when using standardised initial parasitaemia during the assays (clones or isolates), no correlation between CQ and FQ responses were found [22,23]. *In vivo* against *Plasmodium vinckei vinckei*, no antagonism was observed between FQ and artesunate. The survival time of infected mice treated with the association of the two drugs is even increased [24].

*In vivo* experiments performed on different murine *Plasmodium* species showed that the curative dose of FQ (10mg/kg/d for 4 days) remained unchanged, regardless of the susceptibility of the strain to CQ, and the means of administration (subcutaneously or orally); this demonstrates a good availability of the drug and its powerful activity. In addition, the impairment of the host immune responses by FQ was assessed. To this end, the effect on naive animal responses as well as on those of *Plasmodium* infected hosts was evaluated. FQ has shown at least two advantages over CQ. The first is its rapid effectiveness on blood parasites and on the decrease of CD4+CD25+ T-cells. In this context, CD4+CD25+ T cells have been shown to be involved in the expression of susceptibility to experimental malaria infection. However their depletion delayed the growth of parasitemia [25]. Hence, any decrease of these cells would be very beneficial for the expression of protective immunity. Second, its ability to maintain the capacity of spleen cells to proliferate in response to different mitogens [26].Taking into account the absence of any observable immuno-toxicity in rats, together with its efficacy against CQ-resistant strains in vitro, FQ could provide an effective alternative treatment for *P. falciparum* in the future.

Results of both *in vitro* tests on field isolates and *in vivo* experiments on rodent models indicated that a potential resistance to FQ does not depend on a gene polymorphism already involved in CQ resistance. This was shown on Cambodian isolates [22,23] and extended to 15 *P. falciparum* laboratory strains, concerning four genes currently involved in drug resistance (*pfcrt*, *pfmdr1*, *pfmrp*, *pfhne1*) [27]. Pressure experiments were carried out to obtain FQ resistant *P. falciparum* or rodent malaria parasites [9,28]. The results showed that fit-cost of FQ resistance is extremely high, and that surviving parasites exhibit a reduced vitality. Moreover, the resistance observed in rodent malaria parasites appeared not to be genetically integrated [9].

The results of antimalarial measurements and pressure experiments led us to question the origin of the efficacy of FQ. Two principal hypotheses could be envisaged: an absence of interaction with resistance mechanisms already known or a mechanism of action differing from that of CQ and yet to be elucidated.

**Figure 3:**
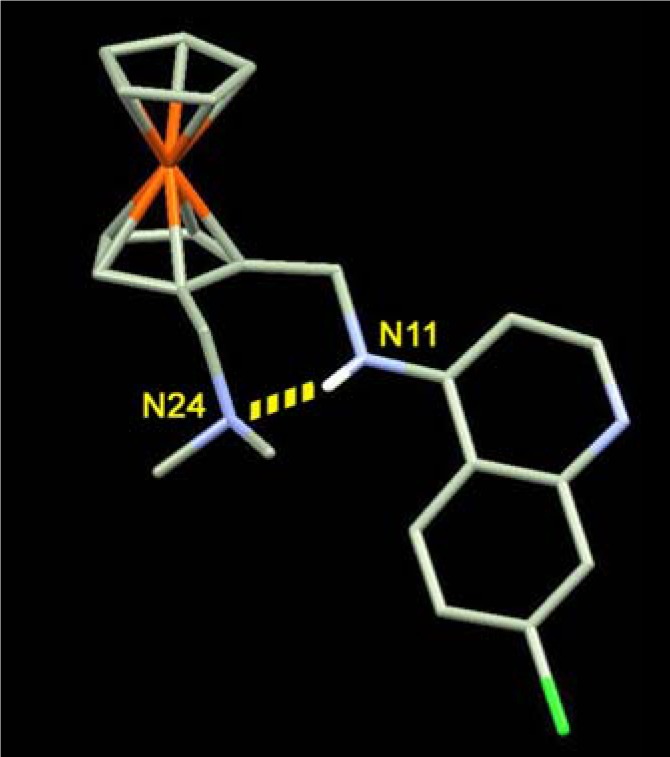
Intramolecular hydrogen-bond in neutral FQ.

Note here that it is necessary to distinguish between the activity on resistant strains (CQ-resistant strains are as sensitive as CQ-susceptible strains to FQ) and the global level of activity. If the first properties are shared by all molecules exhibiting a ferrocene moiety in their lateral chain, between N11 and N24 (Figure 3), and possibly by many molecules incorporating an organic hydrophobic cycle [11], all molecules have different antimalarial activity. The most powerful molecules, as shown in previous studies [9], were those which are able to establish an hydrogen bond between N11 and N25, rendering the molecule more rigid and allowing to the ferrocene core to interact with hydrophobic macromolecules (membranes and lipid bodies) of the DV.

## Mechanism of action

Compared to CQ, the basicity and lipophilicity of FQ are significantly different [29]. When protonated at the putative food vacuole pH of 5.2, the lipophilicity of FQ and CQ are similar (log *D* = –0.77 and –1.2 respectively), whereas they differ markedly at pH 7.4 (log *D* = 2.95 and 0.85 respectively). In addition, the p*K*_a_ values of FQ are lower (p*K*_a1_ = 8.19 and p*K*_a2_ = 6.99) than those of CQ (10.03 and 7.94, respectively). Moreover, these differences in p*K*_a_ values influence the relative concentrations of each microspecies at vacuolar pH. The neutral and monoprotonated forms of FQ are thus 10 times more concentrated at the pH of DV than those of CQ. These forms are those supposed to interact with hematin and its conversion into hemozoin [30]. At cytosolic pH, FQ is 100 times more lipophilic than CQ. Finally, around pH 5, FQ is expected to concentrate 50-fold more than CQ [17]. Given these different behaviours, it was postulated that FQ could target the lipid site of hemozoin formation more efficiently [31]. The solid state structure of neutral FQ is stabilized by a strong intramolecular hydrogen bond between the anilino N(11) and the tertiary amino N(24) (Figure 3). NMR data show that the spatial structure in solution (with a low dielectric constant such as the lipid environment) of FQ is much the same as in the crystal [17]. The role of this non-covalent interaction on the antimalarial activity was questioned. First, the flip/flop H-bond between the open conformation of the charged FQ and the folded conformation of the uncharged FQ should contribute to the transport from water to the hydrophobic membranes. Secondly, a recent study has shown that the analogue bearing a methyl group instead of a hydrogen atom on the anilino N(11), has a markedly reduced activity against both CQ-susceptible and CQ-resistant strains even though its physicochemical properties were practically unaffected [17]. FQ adopts a remarkable conformation in which the side chain is coplanar with the quinoline ring and the bulky ferrocenyl moiety is rejected towards the outside.

**Figure 4:**
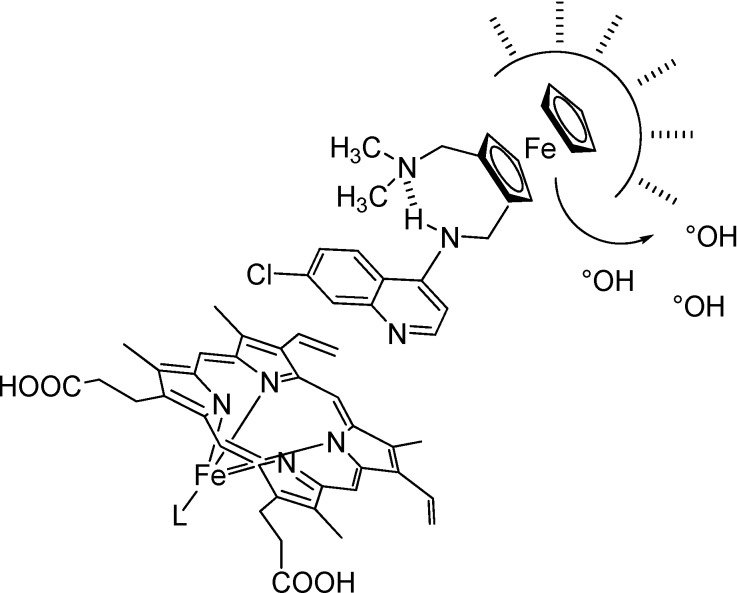
Proposed privileged sites of interaction surrounding FQ.

In contrast to CQ, the flexibility of the side chain of FQ is reduced due to the presence of the rigid ferrocene core, and is linked with an increase in entropy. As observed for peptides [32], this increase of entropy could be one of the main driving forces for clustering of FQ in the lipids of the membrane. A reasonable minimum expectation would be that FQ establishes favorable interaction due to hydrophobic collapse with the lipids of the membrane whereas the quinoline ring is exposed to water (Figure 4).

Like a Trojan horse, FQ can exert its antimalarial activity not only by a mechanism similar to CQ but also by an original mechanism. Like CQ, FQ forms complexes with hematin in aqueous solution (log K = 4.95 ± 0.05). This value is similar to that previously reported for CQ. FQ is a stronger inhibitor of β-hematin formation than CQ. Indeed, the IC_50_ of FQ was 0.8 equivalents relative to hematin, whereas the IC_50_ of CQ was 1.9. An alternative explanation for the highest antimalarial activity of FQ compared to the “classical” organic drugs could be due to its (proposed) preferential localization at the lipid-water interface. FQ could prevent the conversion of hematin into hemozoin by maintaining toxic hematin in the aqueous environment. This hypothesis would explain why the activity of FQ is steady despite the level of resistance of the strains. 

L stands for a molecule of water or another molecule of ferriprotoporpphyrin IX.

Unlike CQ, FQ shows a reversible one-electron redox reaction under the oxidizing conditions of the digestive vacuole of the parasite [33]. Formation of the ferriquinium salt is accompanied by the generation of hydroxyl radicals:

FQ(II) + H_2_O_2_ → FQ(III) + HO^-^ + HO°

Moreover, this production of free radicals in the μM range does not appears sufficient enough to affect the stability of FQ. The accumulation of FQ close to the DV membrane could generate ROS formation and lipid peroxidation (Figure 4). Conjugation of both mechanisms (inhibition of hemozoin formation and ROS generation) leads to the death of the parasite.

## Conclusions

The new organometallic antimalarial drug FQ is extremely active against both CQ-susceptible and CQ-resistant *P. falciparum*. Nevertheless, the cause of activity of FQ on CQ-resistant strains remains totally unexplained. A fifteen year collaborative research program between chemists and biologists offers new clues in our understanding of atypical mechanisms of action in addition to the stated inhibition of hemozoin formation of the quinoline compounds. Briefly: (i) FQ is expected to concentrate 50-fold in the DV more than CQ, (ii) the concentration of active forms of FQ in the DV is 10 times greater than that of CQ, (iii) due to its higher lipophilicity and its shape, FQ should target lipids site more efficiently than CQ, (iV) FQ is a stronger inhibitor of β-hematin formation than CQ, and (v) under the specific conditions (acidic and oxidizing) of the DV, FQ is able to generate ROS whereas CQ is not. Future work will be devoted to studying how FQ acts against CQ-resistant strains. Particularly, generation of ROS *in situ* and determination of malondialdehyde (MDA) as a marker of lipid peroxidation. The studies will also aim to investigate in detail the interaction between FQ and lipids.
